# A Unified Taxonomy for the Circulating Tumor Microenvironment (cTME) and Circulating Tumor-Associated Cells (C-TACs): A Conceptual Framework for Precision Oncology

**DOI:** 10.3390/cells15121108

**Published:** 2026-06-18

**Authors:** Noriyoshi Sawabata

**Affiliations:** 1 Department of Thoracic Surgery, Kawanishi City Medical Center, Kawanishi 666-0017, Japan; nsawabata@hotmail.com; Tel.: +81-570-01-8199; 2 Department of Diagnostic Pathology, Nara Medical University, Kashihara 634-8521, Japan

**Keywords:** liquid biopsy, circulating tumor microenvironment (cTME), circulating rare cells (CRCs), circulating tumor-associated cells (C-TACs), circulating tumor cells (CTCs), circulating tumor emboli (CTE), circulating tumor endothelial cells (CTECs), next-generation sequencing (NGS), chemo-response profiling (CRP), precision oncology, taxonomy, metastasis

## Abstract

**Simple Summary:**

As liquid biopsy evolves into a multi-omic platform, the growing diversity of circulating tumor entities demands a structured classification framework. We propose a multi-tiered taxonomy: cTME as the superordinate systemic ecosystem encompassing cellular, non-cellular, and biophysical components; CTE for physical multicellular aggregates; and C-TACs for the functional cellular units that execute metastasis. This hierarchical framework enables clinicians to systematically integrate molecular profiling with functional cellular assays, with the potential to develop liquid biopsy into a precision oncology tool for predicting adjuvant chemotherapy efficacy and guiding personalized cancer care.

**Highlights:**

**What are the main findings?**

- A unified hierarchical taxonomy was established to decouple the systemic “Circulating Tumor Microenvironment” (cTME) from aggressive physical aggregates termed “Circulating Tumor Emboli” (CTE).
- Circulating Tumor-Associated Cells (C-TACs) are proposed as the primary functional cellular executors, with proof-of-concept evidence of up to 97% concordance with radiological treatment responses through Chemo-Response Profiling (CRP), pending independent prospective validation.

**What are the implication of the main findings?**

- This structured taxonomy provides a standardized framework for clinical reporting and enables the seamless integration of multi-modal liquid biopsy data across diverse technological platforms.
- The identification of functional units, specifically perioperative CTE, may provide a candidate roadmap for predicting adjuvant chemotherapy efficacy and informing personalized cancer management in thoracic oncology, pending prospective validation.

**Abstract:**

Background: The growing complexity of liquid biopsy in precision oncology demands a structured classification framework that can accommodate its expanding multi-omic scope. As the field has matured from early Tumor Microemboli research—focused on multicellular clusters of circulating tumor cells (CTCs) that drive high-efficiency metastasis—to the broader systemic analysis of the “Tumor Microenvironment” (TME) encompassing malignant and non-malignant components, the need for a hierarchical taxonomy has become evident. Objective: To integrate these diverse data streams into a coherent clinical framework, a multi-tiered classification system is needed. This review proposes a foundational roadmap that formally distinguishes the systemic ecosystem from its physical and functional subsets and highlights their clinical utility in therapeutic decision-making. Proposed Taxonomy: We advocate for the adoption of Circulating Tumor Microenvironment (cTME) as the inclusive term for the systemic environment, encompassing non-cellular factors such as ctDNA, extracellular vesicles, and biophysical attributes. Conversely, physical cellular clusters should be strictly classified as Circulating Tumor Emboli (CTE). Crucially, we define Circulating Tumor-Associated Cells (C-TACs) as the functional cellular subset within the cTME, encompassing single CTCs, CTE, and supporting non-malignant cells like CTECs and CAFs. Clinical Applications: Establishing this distinction allows for the seamless integration of molecular profiling (NGS) and functional assays. We highlight emerging evidence that C-TACs may serve as the primary substrate for Chemo-Response Profiling (CRP), with early proof-of-concept studies reporting high concordance with clinical outcomes that still await independent prospective confirmation. Furthermore, preliminary evidence suggests that identifying these functional units, particularly perioperative CTE, may help predict the efficacy of adjuvant chemotherapy in early-stage malignancies, although this remains to be confirmed in prospective studies. Conclusions: Adopting this unified taxonomy may help advance precision oncology. By recognizing the cTME as the superordinate ecosystem and C-TACs as its functional executors, clinicians may be better positioned to interpret multi-modal liquid biopsy data, providing a conceptual roadmap for integrating these technologies into platforms for personalized cancer management. We emphasize that this framework is intended to be hypothesis-generating and that its clinical applications require prospective validation before routine adoption.

## 1. Introduction: The Metastatic Cascade and the Need for a Structured Taxonomy

The understanding and control of the metastatic cascade remain the most formidable challenges in modern oncology, as this complex biological process is responsible for more than 90% of cancer-related fatalities [1,2,3]. In the clinical setting, the ability to intercept the metastatic process at its earliest stages represents the “holy grail” of precision medicine [4]. Over the past decade, liquid biopsy has emerged as a revolutionary, non-invasive paradigm that offers an unprecedented window into the real-time molecular and cellular evolution of tumors [5,6,7]. As the field has matured, the analytical scope has transcended simple cell enumeration—once limited to the counting of individual circulating tumor cells (CTCs) [8,9]—to encompass a sophisticated and dynamic ecosystem of multi-modal biomarkers, including circulating tumor DNA (ctDNA) and extracellular vesicles (EVs) [10,11,12].

However, as we stand on the threshold of integrating these multi-omic insights into routine clinical practice, the need for a structured, hierarchical classification framework has become increasingly apparent [13]. This is not merely a semantic concern; it is a structural gap that compromises the standardized reporting of clinical results and the comparability of research findings across different technological platforms [13,14]. A central motivation for a structured taxonomy is the need to formally distinguish the diverse biological entities that coexist within the circulating tumor landscape [1].

For decades, clinical pathology and early metastasis research have heavily focused on Tumor Microemboli—the physical, multicellular aggregates or clusters of tumor cells that drive aggressive metastasis with a seeding efficiency 23 to 50 times higher than that of solitary cells [15,16,17,18,19]. A seminal study by Hou et al. [20] demonstrated the profound clinical significance of these circulating tumor microemboli in small-cell lung cancer, emphasizing their role as a distinct biomarker from single CTCs. Furthermore, the term “microemboli” remains an active clinical descriptor, as evidenced by recent research in this journal by Chu et al. [21], who utilized it to distinguish aggregates in colorectal cancer prognosis. Conversely, in contemporary cancer biology and systemic therapy research, “TME” has become the universal shorthand for the Tumor Microenvironment, representing the complex systemic landscape of malignant cells, immune components, and the extracellular matrix [1,2,15]. As the field integrates multi-omic and ecosystem-based data, a formal classification that distinguishes physical cellular aggregates from the broader biological landscape of the TME is essential for standardized clinical application [1,13].

This lack of clarity is particularly problematic when integrating functional and genomic data [13]. For instance, while next-generation sequencing (NGS) of ctDNA provides critical insights into clonal evolution, genomic data alone often fails to capture the dynamic drug resistance profiles of the functional cellular executors [21,22]. Furthermore, the boundaries between solitary CTCs—including recently identified “small-size” variants (<5 μm) that serve as distinct prognostic indicators—and physical aggregates (CTE) have become increasingly blurred [19,23,24].

In this review, we propose a rigorous taxonomic hierarchy designed to accommodate the growing complexity of liquid biopsy in the era of precision oncology [4,12,13]. We advocate for the formal adoption of the Circulating Tumor Microenvironment (cTME) as the superordinate hierarchical tier, representing the inclusive systemic ecosystem [1,4]. Within this framework, Circulating Tumor-Associated Cells (C-TACs) are defined as the primary functional cellular executors—heterogeneous collectives that encompass malignant cells and their supporting non-malignant partners, such as circulating tumor-derived endothelial cells (CTECs) and cancer-associated fibroblasts (CAFs) [24,25,26,27]. By strictly decoupling the systemic environment (cTME) from the physical emboli (CTE), this framework provides a robust roadmap for personalizing real-time cancer management, particularly in predicting the efficacy of adjuvant therapies and monitoring minimal residual disease [1,12,19,27].

## 2. Materials and Methods: PRISMA-Guided Synthesis of the Circulating Ecosystem

To establish a rigorous and evidence-based taxonomic framework, we conducted a systematic synthesis of the existing literature following the PRISMA (Preferred Reporting Items for Systematic Reviews and Meta-Analyses) guidelines [14]. This methodological choice was intentional, ensuring that the proposed nomenclature is not merely an anecdotal suggestion but is grounded in a comprehensive analysis of the evolving liquid biopsy landscape over the past decade [4,5,13].

### 2.1. Search Strategy and Data Acquisition

We performed an extensive longitudinal search across the PubMed, Scopus, and Google Scholar databases, covering the period from January 2010 to January 2026 [4,6,13]. This timeframe was selected to capture the critical transition from early circulating tumor cell (CTC) enumeration studies to the current era of multi-modal, ecosystem-based profiling.

The search strategy was designed to identify the linguistic origins of current terminological confusion while simultaneously gathering clinical evidence for new classifications. Primary keywords and Boolean operators included:

- (“circulating tumor microenvironment” OR “cTME”) [1];
- (“circulating tumor-associated cells” OR “C-TACs”) [24];
- (“liquid biopsy”) [5,13];
- (“taxonomy” OR “nomenclature”) [13,14].

To ensure the clinical relevance of our proposed hierarchy, secondary searches were focused on specific functional outcomes and validation metrics. These included terms such as “chemo-response profiling (CRP)”, “97% concordance”, “adjuvant chemotherapy efficacy”, and the biological concept of the “mobile niche” [1,19,28].

### 2.2. Eligibility Criteria and Study Selection

**Figure 1:**
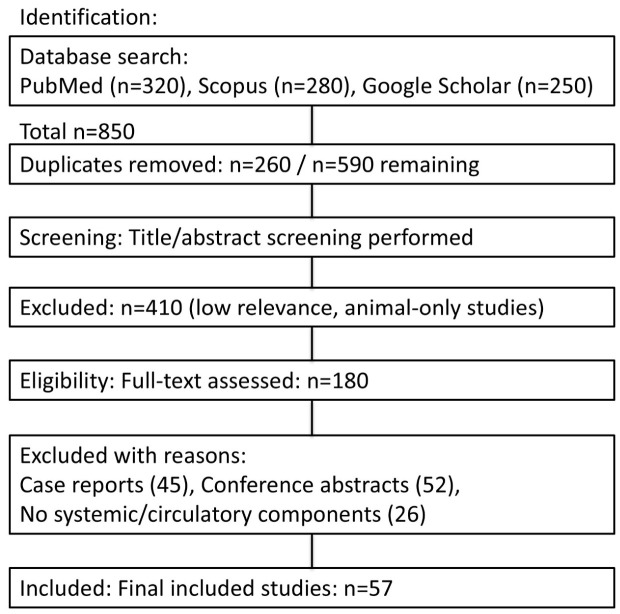
PRISMA flow diagram of the study selection process.

A total of 850 records were initially identified. After removing 260 duplicates, 590 records were screened by title and abstract. 180 full-text articles were assessed for eligibility. Studies were included if they provided explicit definitions for circulating tumor components or clinical evidence regarding the predictive value of CTE or C-TACs. We strictly excluded case reports, conference abstracts, and studies focused exclusively on the localized primary tumor microenvironment. Finally, 57 high-impact publications—including meta-analyses and prospective clinical trials—were selected for final synthesis (Figure 1). We prioritized studies that provided distinct analytical thresholds for therapeutic decision-making [29,30,31,32,33,34]. Of note, these 57 publications constitute the studies identified and selected through the systematic search described above. A limited number of additional references were cited elsewhere to provide essential conceptual background, to support the methodological framework (e.g., the PRISMA statement), or in response to peer-review recommendations; such contextual citations did not enter the systematic screening corpus and are therefore not reflected in the PRISMA flow counts.

The diagram summarizes the identification, screening, and inclusion phases of the systematic literature review conducted to establish the cTME/C-TAC taxonomy. A total of 850 records were initially identified from PubMed (n = 320), Scopus (n = 280), and Google Scholar (n = 250). After removing 260 duplicates, 590 unique records were screened by title and abstract, leading to the exclusion of 410 records due to low relevance or animal-only focus. Full-text assessment was performed on 180 articles, of which 123 were excluded for specific reasons: case reports (n = 45), conference abstracts (n = 52), and studies lacking a systemic or circulatory component (n = 26). Ultimately, 57 high-impact publications were included in the final qualitative synthesis.

### 2.3. Data Synthesis, Qualitative Analysis, and Hierarchical Mapping

The proposed taxonomy was formulated by mapping these synthesized findings into a hierarchical biological structure. Findings from historical pathology studies focusing on tumor microemboli and modern molecular biology focusing on the systemic tumor microenvironment (TME) were cross-referenced to establish clear categorical boundaries within the circulating tumor landscape [15,16,18,19]. This qualitative synthesis allowed us to define the functional boundaries between the systemic ecosystem (cTME) and its cellular executors (C-TACs), providing the evidence-based foundation for the chapters that follow [12,13]. During the preparation of this manuscript, the author used Claude Opus 4.8 (Anthropic) to assist with English-language editing and to verify the internal consistency of the reference list. The author has reviewed and edited all output and takes full responsibility for the content of this publication.

## 3. The Hierarchical Taxonomy: Defining the cTME as a Systemic Ecosystem

The core of our proposal lies in the establishment of a rigorous, multi-tiered hierarchy that accurately reflects the biological complexity of the circulating tumor landscape [4,12,13]. To operate this system, we must first formally distinguish the systemic ecosystem from its physical cellular subsets. Historically, clinical pathology and early-stage metastasis research focused on Tumor Microemboli—defined as the physical, multicellular aggregates or clusters of tumor cells that exhibit significantly enhanced metastatic potential [9,17,18,19,24]. However, contemporary cancer biology has rightfully transitioned “TME” into the universal designation for the Tumor Microenvironment, representing the vast systemic landscape of malignant cells, immune components, and the extracellular matrix [1,2,31].

Building upon this transition, we advocate for the formal adoption of the Circulating Tumor Microenvironment (cTME) as the superordinate tier of our taxonomic framework [1,4]. The cTME is not merely a transport medium; it is a dynamic, “flowing” microenvironment that differs fundamentally from the localized primary or metastatic TME [1,3]. This systemic perspective necessitates a classification system that integrates cellular, non-cellular, and biophysical attributes into a single cohesive ecosystem [8,11,12].

### 3.1. The Conceptual Superordinate Tier: cTME

The cTME represents the highest hierarchical tier, serving as the inclusive systemic environment within the peripheral blood and other body fluids [1,4]. This superordinate classification is critical because it acknowledges that the fate of a circulating tumor cell is not determined in isolation but is governed by a complex array of systemic regulators [1,3]. By designating the cTME as the “superordinate tier,” we provide a conceptual umbrella that encompasses both the functional cellular executors (C-TACs) and the molecular instructions that prepare distant organs for colonization [1,24,35,36].

### 3.2. Non-Cellular Components: Molecular Messengers and Genomic Blueprints

A key feature distinguishing the cTME from its cellular subsets is the inclusion of non-cellular components, which may be conceptualized as the “software” of the metastatic process [1,5,11,36].

- Circulating Tumor DNA (ctDNA) and NGS: ctDNA serves as a critical genomic blueprint within the cTME [1,12]. Through the application of Next-Generation Sequencing (NGS), clinicians can detect somatic mutations, copy number variations, and patterns of clonal evolution that occur in response to therapeutic pressure [23,36,37,38]. While ctDNA provides a high-resolution map of the tumor’s molecular identity, it must be viewed as one component of the broader cTME ecosystem [1,12].
- Extracellular Vesicles (EVs) and the Pre-Metastatic Niche: EVs, particularly exosomes (30–150 nm), are structurally distinct from cellular units as they are lipid-bilayer-enclosed particles lacking a functional nucleus [37,38]. Within the cTME, EVs function as “molecular fertilizers” that actively reprogram distant organs to establish Pre-Metastatic Niches (PMNs) [11,36]. This preparatory function allows the cTME to influence organs long before the arrival of physical tumor cells [35,36].
- Advanced Enrichment via pH Sensitivity: Recent technological advancements, such as the D-S PHLIP (double-switch pH-low insertion peptide) system, have revolutionized our ability to study these messengers. By exploiting the localized acidic environment associated with systemic tumor activity—often reaching a pH ≈ 6.8—these systems allow for the selective enrichment of cTME-derived EVs, providing a clearer window into the preparatory signals of metastasis [39].
- Fragmentomics, Methylation, and Systemic Epigenetics: Beyond conventional somatic mutation calling, the non-cellular landscape of the cTME now encompasses fragmentomics—including cell-free DNA fragment-end motifs, nucleosome positioning, and size-distribution profiles—and cell-free DNA methylation profiles, which have emerged as highly sensitive biomarkers for multi-cancer early detection and tissue-of-origin prediction [40].

### 3.3. Biophysical Regulators: The Mechanical Environment

Equally significant to the molecular components are the biophysical attributes that define the cTME [3,41]. The peripheral circulation is a hostile mechanical environment, and the survival of a tumor cell is contingent upon its ability to navigate these forces [3,26,41].

- Fluid Shear Stress (FSS): The FSS exerted by blood flow acts as a powerful selective pressure, eliminating vulnerable cells while favoring those with mesenchymal or clustered phenotypes [3,26,41].
- Flow Velocity and Capillary Entrapment: The velocity of blood flow and the physical dimensions of capillary beds determine the likelihood of tumor cell arrest and subsequent extravasation [3,16].
- Systemic Acidic pH: Beyond localized tumor sites, the systemic blood associated with aggressive malignancies can exhibit an acidic shift (pH ≈ 6.8) [39], which not only serves as a biomarker but also influences the membrane stability and signaling efficacy of both cells and vesicles within the cTME [39].

**Figure 2:**
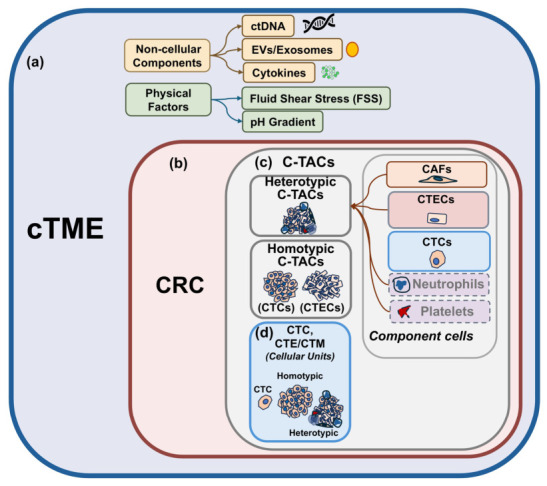
Simplified conceptual overview of the cTME hierarchy and functional cell taxonomy.

By strictly defining the cTME through these three pillars—cellular subsets, non-cellular messengers, and biophysical regulators—we establish a structured framework that provides the necessary clarity for the next generation of precision oncology [1,4,12,13]. The organizational complexity of this systemic ecosystem, structured according to these defined pillars, is visually integrated into the hierarchical framework illustrated in Figure 2.

This figure provides a conceptual overview of the proposed unified taxonomy, organized as a set of nested hierarchical levels. Panel (a) shows the non-cellular components (ctDNA, EVs/exosomes, cytokines) and physical factors (fluid shear stress, pH gradient) that operate at the level of the circulating tumor microenvironment (cTME). Panel (b) denotes the Circulating Rare Cells (CRC) compartment. Panel (c) shows the Circulating Tumor-Associated Cells (C-TACs), comprising heterotypic and homotypic assemblies together with their component cells (CAFs, CTECs, CTCs, neutrophils, and platelets). Panel (d) shows the elemental cellular units (CTC and CTE/CTM, in homotypic and heterotypic configurations). To keep the diagram readable, detailed functional roles and clinical associations are not labelled within the figure but are described in the legend below.

(a) Circulating Tumor Microenvironment (cTME): The inclusive superordinate ecosystem governed by non-cellular components (ctDNA, EVs, cytokines), biophysical factors (FSS, pH), and systemic inter-cellular communication [1,3,4,10,11,12].

(b) Circulating Rare Cells (CRC): The broadest cellular category, serving as the primary diagnostic umbrella for all non-hematological rare events found in circulation [1,13,24,42].

(c) C-TACs (Circulating Tumor-Associated Cells): The functional unit of the cTME, representing the “mobile niche” [1,12,15,24].

- Component cells: C-TACs are heterogeneous assemblies in which malignant Circulating Tumor Cells (CTCs) constitute the core, supported by host-derived partner cells. CAFs (Cancer-Associated Fibroblasts) confer shear resistance and are central to the EpCAM paradox [26,27,43,44,45]. CTECs (Aneuploid Circulating Tumor-Derived Endothelial Cells) facilitate vascular mimicry and adhesion [23,25,41]. Neutrophils act as escorts via inflammation-mediated homing [46], and Platelets provide a protective shield for immune evasion and survival [1,47].

(d) CTC, CTE/CTM (Cellular Units): The physical manifestation of metastasis. Individual malignant cells (CTCs) and multicellular aggregates (CTE/CTM) serve as the elemental executors of the metastatic cascade. CTE are strictly distinguished from solitary cells due to their 23 to 50 times higher metastatic potential [18,19,20,21,48].

Clinical Relevance: The proposed taxonomy is intended to link biological profiling with potential clinical applications. In a proof-of-concept study, functional assays utilizing the C-TAC collective reported a 97% CRP concordance with treatment response [28]. In addition, preliminary evidence suggests that the identification of clustered units, specifically CTE, may serve as a candidate predictor of adjuvant chemotherapy efficacy in perioperative lung cancer management [19]. These associations are derived from a limited number of studies and require independent prospective validation. For clarity, detailed functional roles of the component cells (CAFs, shear resistance and the EpCAM paradox; CTECs, vascular mimicry and adhesion; neutrophils, inflammation-mediated homing; platelets, immune evasion and survival) are described in panel (C) above rather than within the diagram.

Color codes and symbols: (a) The light blue background represents the overall circulating tumor microenvironment (cTME), including non-cellular components (ctDNA, DNA symbol; EVs/exosomes, yellow circle; cytokines, green dots) and physical factors. (b) The light red solid box delineates circulating rare cells (CRCs). (c, d) Component cells within the clusters are color-coded as follows: CAFs (orange), CTECs (pink), CTCs (blue), and immune cells/platelets (purple dashed boxes). Homotypic and heterotypic clusters are illustrated based on these cellular components.

## 4. C-TACs: The Functional Cellular Unit for Therapeutic Prediction

The transition from a descriptive liquid biopsy to a functional diagnostic platform necessitates an ontological shift in how we define the primary cellular executors of the metastatic cascade [5,13]. For decades, the focus was centered exclusively on the solitary Circulating Tumor Cell (CTC)—the individual malignant “seed” shed from a primary or metastatic lesion [41,49]. While CTC enumeration (e.g., via the CellSearch^®^ system) provided foundational prognostic insights [32,41,49,50], it failed to capture the biological complexity of the “flowing microenvironment” [3,4]. Within our proposed hierarchical framework, we introduce Circulating Tumor-Associated Cells (C-TACs) as the superordinate cellular tier, representing the functional cellular subset of the broader cTME [12,24].

### 4.1. From “Single Seeds” to the “Mobile Niche”

The term “C-TACs” represents a fundamental paradigm shift: viewing metastasis not as an isolated single-cell event, but as a coordinated, collective process facilitated by a heterogeneous ensemble of malignant and non-malignant partners [12,15,17,24,51]. Unlike traditional assays that prioritize the isolation of solitary malignant cells, the C-TAC framework recognizes that the physical execution of metastasis is carried out by a “mobile niche”—a functional ecosystem [1] that remains operationally intact even while in transit through the systemic circulation [1,3].

This ensemble is the primary substrate for precision diagnostics because it reflects the real-time resistance profile and therapeutic sensitivity of the disease more accurately than isolated genomic data alone [9,12,28]. The C-TAC population functions as the active “executor” of metastasis, shielding the malignant core from environmental stressors and preparing the distant site for colonization [1,26,51].

### 4.2. Deep Dive into the Cellular Composition of C-TACs

The functional potency of C-TACs is derived from the synergistic interactions between various rare cellular subpopulations. These include:

- Circulating Tumor-Derived Endothelial Cells (CTECs): A critical, yet frequently overlooked, component of the C-TAC framework is the CTEC [25,52]. These are tumor-derived endothelial cells that exhibit significant genetic abnormalities, such as aneuploidy, which mirror the chromosomal instability of the primary tumor’s neovasculature [25]. Within the C-TAC collective, CTECs facilitate CTC survival by maintaining pro-angiogenic signaling and protecting the malignant core from immune surveillance [25,52].
- Cancer-Associated Fibroblasts (CAFs): These cells migrate from the primary tumor stroma and enter the circulation alongside CTCs [26,27]. CAFs are essential for conferring resistance to fluid shear stress (FSS) and promoting the motility required for extravasation at distant sites [24,25,26].
- Immune “Escorts” (Neutrophils and Macrophages): Metastatic efficiency is significantly enhanced by the interaction between CTCs and systemic immune cells [1,46,51]. Specifically, neutrophils have been shown to “escort” CTCs via VCAM-1-dependent intercellular junctions [46]. This physical interaction facilitates cell cycle progression—often evidenced by Ki67 overexpression—within the C-TAC cluster, thereby increasing the likelihood of successful seeding [46,51].
- Platelets and “Immune Cloaking”: Through a process known as tumor cell-induced platelet aggregation (TCIPA), platelets associate with C-TACs to form a protective “cloak” [26,47]. This biological shield masks the malignant cells from natural killer (NK) cells and other executors of the innate immune system [1,47].

### 4.3. Preliminary Clinical Evidence: The 97% CRP Concordance

One supporting argument for considering C-TACs as a primary taxonomic unit for cellular liquid biopsy is the emerging, though still preliminary, evidence of their utility in therapeutic prediction [24,28,53]. Previous attempts to correlate chemo-response profiling (CRP) based on solitary CTCs with radiological outcomes were largely unsuccessful due to the loss of functional context [4,13].

However, when CRP is performed using the complete C-TAC collective, research by Crook et al. [28] reported a 97% concordance with radiological treatment responses in therapy-naïve patients. In pretreated patients, this concordance was lower at 87%, which may reflect functional adaptations (“resistance education”) induced by prior systemic therapy. Taken together, these preliminary observations are consistent with the possibility that the C-TAC framework could provide a real-time readout of the disease’s functional state, although this interpretation requires confirmation in independent prospective cohorts [28]. While these findings should be regarded as highly encouraging—as large-scale, proof-of-concept evidence that still requires further prospective standardization—they strongly support the use of the C-TAC ensemble as a real-time functional readout of the disease. It should be emphasized that these conclusions—the high concordance, the designation of C-TACs as the principal functional unit, and the use of perioperative CTE to guide adjuvant therapy—currently rest on a limited number of studies, including our own, several of which are single-centre or retrospective in design. Independent, multi-centre, prospectively designed validation will therefore be essential before these findings can be regarded as established, and we present this framework as a hypothesis-generating roadmap rather than as a settled clinical standard.

### 4.4. Implications for Perioperative Management in Lung Cancer

Furthermore, the practical utility of this framework is evidenced in early-stage disease management, particularly in Non-Small Cell Lung Cancer (NSCLC) [4,19]. Perioperative identification of clustered C-TAC units (specifically those categorized as CTE) may serve as a candidate predictor of adjuvant chemotherapy efficacy [19]. Patients harboring these functional units show a significant improvement in 2-year recurrence-free survival (RFS) when treated with adjuvant therapy—71.8% compared to only 36.3% in those without adjuvant treatment [19]. This degree of clinical predictability, if confirmed, would be consistent with the notion that C-TACs may help isolate a “functional cellular unit” relevant to drug sensitivity [19,24].

By defining C-TACs as the functional executors within the systemic ecosystem of the cTME [1,24], we move beyond the limitations of simple cell enumeration [4,13] and establish a clear roadmap for the integration of functional cellular data into personalized cancer management [12,13].

## 5. Aggressive Aggregates: The Biology and Seeding Potential of CTE and CTM

The most formidable physical entity within the circulating tumor landscape is the multicellular aggregate, which must be strictly and uniformly termed Circulating Tumor Emboli (CTE) or Circulating Tumor Microemboli (CTM) [18,19,21]. As the field advances toward multi-modal profiling, the formal distinction between these physical aggregates and the broader systemic microenvironment (TME) is essential, and this distinction forms a central pillar of the proposed taxonomic hierarchy [1,13]. Within the proposed hierarchical framework, CTE represent a critical, highly aggressive subset of the C-TAC population—the physical manifestation of the “mobile niche” required for successful distal colonization [1,21,24].

### 5.1. The Biomechanical Advantage: Superior Metastatic Efficiency

The biological significance of CTE lies in their disproportionate contribution to the metastatic process [15,16,19]. Despite representing a minority of the total circulating tumor burden, these aggregates possess a metastatic seeding efficiency estimated to be 23 to 50 times higher than that of solitary CTCs [18]. This pronounced advantage is thought to reflect not merely cell number, but emergent biomechanical and survival properties associated with the clustered state [3,15,26].

- Resistance to Anoikis: Solitary CTCs are highly susceptible to anoikis—a form of programmed cell death triggered by the loss of cell–matrix or cell–cell interaction [16,18]. Within a CTE, however, the dense network of intercellular junctions, facilitated by cadherins and other adhesion molecules, provides a constant pro-survival signal that mimics the architecture of the primary tumor [17,18,51,54]. This internal support system effectively bypasses the apoptotic triggers that typically eliminate individual cells in transit [18,26].
- Hydrodynamic Protection from Shear Stress: The peripheral circulation is a hostile environment defined by high-velocity blood flow and intense Fluid Shear Stress (FSS) [3,16,26,41]. Individual cells face physical fragmentation or mechanical trauma; in contrast, the aggregate structure of CTE shields the central malignant cells from these forces [3,16,18,26]. By providing a “physical shield,” the outer layers of the cluster absorb the kinetic energy of the flow, preserving the viability and proliferative capacity of the inner core [3,18,26].
- Facilitated Capillary Entrapment: Due to their larger physical dimensions, CTE are significantly more likely than single CTCs to become mechanically trapped within the narrow capillary beds of distant organs [3,16]. This entrapment is not merely a passive blockage but serves as the initiating event for extravasation, allowing the cluster to seed the “soil” of the distant site with high cell density [3,16,18].

### 5.2. Homotypic vs. Heterotypic Clusters: The Complex Ecosystem

To achieve a “Total cTME Profiling,” we must distinguish between the composition of these aggregates [1,12,24,51].

- Homotypic CTE: Composed exclusively of a single cellular phenotype (e.g., pure malignant tumor cells or pure CTECs), these clusters represent the primary invasive units [17,18,54].
- Heterotypic CTE: These represent the true functional peak of the C-TAC framework, incorporating non-malignant supporting cells such as CTECs [25], CAFs [26,27], and immune cells [24,51]. The presence of host cells within the cluster—such as neutrophils that “escort” the tumor cells—further enhances the cluster’s ability to resist immune surveillance and navigate the metastatic journey [46,47,51].

Through the interaction with platelets (TCIPA), heterotypic CTEs can effectively “cloak” themselves, avoiding detection by natural killer (NK) cells and other immune executors [26,47]. This sophisticated “mobile microenvironment” allows the tumor to transport its own supporting stroma to the secondary site, significantly increasing the probability of successful outgrowth into a macro-metastasis [1,18,24,51].

### 5.3. Clinical Utility: Predictive Power in Lung Cancer Management

The clinical imperative for strictly defining CTE as an aggressive subset is best demonstrated in the perioperative management of Non-Small Cell Lung Cancer (NSCLC) [4,19]. Recent evidence suggests that the perioperative identification of these heterotypic aggregates may help predict the efficacy of adjuvant chemotherapy, although this finding derives from a limited number of studies and awaits prospective confirmation [19].

- In patients in whom CTE were detected (CTE-positive), the administration of adjuvant therapy was associated with a 71.8% 2-year Recurrence-Free Survival (RFS), compared with 36.3% in untreated patients harboring similar clusters [19].
- Conversely, CTE-negative patients did not appear to derive a statistically significant benefit from systemic therapy in this cohort [19].

These observations suggest that CTE may be more than biological curiosities and could represent active contributors to recurrence that warrant further evaluation within the precision oncology roadmap [12,13,19]. By strictly decoupling these aggregates from the systemic environment of the cTME, clinicians can more accurately risk-stratify patients and personalize the intensity of perioperative care [1,10,13,19].

## 6. Architectural Deep Dive: Analyzing the Hierarchical Framework (Table 1)

The proposed taxonomy represents a departure from traditional, flat classification systems that have historically failed to capture the multi-dimensional nature of cancer dissemination [4,13]. By structuring the circulating tumor ecosystem into a five-tier hierarchy, as detailed in Table 1, we provide a functional roadmap that aligns biological complexity with clinical utility [1,13,24]. This architectural approach ensures that each biological entity is defined not merely by its presence, but by its operational scope and its specific contribution to the metastatic cascade [1,18].

### 6.1. The Superordinate Tier: Circulating Tumor Microenvironment (cTME)

The cTME functions as the highest inclusive concept within our taxonomy [1]. It is defined as a holistic system that integrates cellular, non-cellular, and biophysical attributes [1,13]. The scope of the cTME transcends the simple presence of malignant cells; it represents the overall “control center” of metastasis, responsible for the preparation of the Pre-Metastatic Niche (PMN) [1,36]. Within this superordinate tier, non-cellular molecular messengers—such as extracellular vesicles (EVs) and circulating tumor DNA (ctDNA)—function as systemic regulators that “reprogram” distant organs long before the arrival of physical tumor cells [1,11,36,55,56]. By recognizing the cTME as a superordinate ecosystem, we allow for the integration of genomic data (NGS) and biophysical parameters (e.g., pH ≈ 6.8 and fluid shear stress) into a unified prognostic framework [1,3,23,26,41].

### 6.2. The Broadest Cellular Category: Circulating Rare Cells (CRC)

The CRC category serves as the highest cellular tier, encompassing the total breadth of rare circulating populations [13,24,49]. This classification is intentionally broad, providing a diagnostic umbrella for all non-hematological cells found in the circulation [13,49]. The scope of CRCs includes not only malignant tumor cells and their associated partners but also other rare host cells, such as megakaryocytes, circulating endothelial cells, and various macrophage subsets [1,24,25]. In clinical practice, the CRC tier represents the primary screening and detection phase of liquid biopsy, where marker-independent technologies are utilized to capture the entire spectrum of rare events before secondary functional sub-classification [4,13].

### 6.3. The Functional Execution Tier: Circulating Tumor-Associated Cells (C-TACs)

Defining the C-TACs as a functional subset is perhaps the most critical distinction in our taxonomy [1,24]. While the CRC tier identifies all rare cells, the C-TAC tier focuses exclusively on the cellular ensemble responsible for the physical execution of metastasis and therapeutic resistance [1,18,24]. This subset is defined as a heterogeneous collective, incorporating both malignant cells (CTCs) and their essential non-malignant “escorts,” such as CAFs and CTECs [24,25,26,27,51]. The core functional role of C-TACs is to provide the cellular substrate for Chemo-Response Profiling (CRP) [28,53]. As highlighted in Table 1, the clinical focus of C-TACs is their 97% concordance with treatment response, establishing them as the primary unit for functional precision oncology [12,28].

### 6.4. The Aggressive Aggregate Subset: Circulating Tumor Emboli (CTE/CTM)

Within the C-TAC population, multicellular aggregates are strictly classified as CTE or CTM. This category represents an aggressive subset characterized by superior survival and high-efficiency metastatic seeding [3,16,19]. The operational definition of CTE emphasizes their role as a “mobile niche,” where the proximity of cells within the cluster enhances the collective metastatic potential by 23 to 50 times compared to solitary cells [18,54]. Clinically, the identification of CTE represents a high-risk stratification indicator, particularly useful for predicting the recurrence-free survival (RFS) benefit of adjuvant chemotherapy [19].

### 6.5. The Elemental Unit: Circulating Tumor Cells (CTCs)

The base of our hierarchical pyramid is the CTC, defined as the malignant cell in its most elemental form [43,49]. This category includes single malignant cells and homotypic clusters that serve as the primary “seeds” of metastasis [16,18,49]. While CTC enumeration remains the minimum prognostic indicator and the primary target for established platforms like CellSearch^®^, it must be understood as the most basic unit within a much larger and more complex system [1,4,13,41]. Our taxonomy accommodates phenotypic diversity within this tier, including “small-size” CTCs (<5 μm) and cells undergoing epithelial–mesenchymal transition (EMT) [42,43,44,48].

By detailing these hierarchical relationships in Table 1, we provide a structured framework that formally distinguishes each level of the circulating tumor landscape [1,4]. This structure allows clinicians to move seamlessly between broad genomic cues (cTME), functional cellular dynamics (C-TACs), and physical risk markers (CTE), ultimately realizing the vision of truly personalized cancer management [1,12,13,19,24].

## 7. Synthesis of Clinical Foundations: Empirical Validation of the C-TAC/CTE Taxonomy

The theoretical elegance of a taxonomic framework remains hollow unless it is underpinned by rigorous empirical substantiation that translates biological observations into actionable clinical insights. To this end, Table 2 serves as the empirical bedrock of our proposed hierarchy, synthesizing high-impact clinical data that validate the transition from simple cell enumeration to functional and structural profiling. Each study selected for this synthesis represents a pivotal milestone in our understanding of how the circulating tumor ecosystem influences therapeutic outcomes and patient stratification.

### 7.1. Validating the Functional Unity: The Multi-Center Impact of C-TACs

The most significant challenge in personalizing cancer treatment is the high rate of discordance between static genomic maps and real-time clinical responses [4,13,37,38]. The research conducted by Crook et al. across a massive cohort of over 5000 patients profoundly alters this trajectory by validating C-TACs as the primary functional cellular unit [24,28].

- The 97% Concordance Milestone: In therapy-naïve patients, the study demonstrated that Chemo-Response Profiling (CRP) performed on isolated C-TAC collectives achieved a 97% concordance with radiological treatment responses [28]. This level of concordance, while preliminary, is compatible with the hypothesis that the C-TAC ensemble—inclusive of its supporting stroma and immune escorts—may retain functional features of the tumor while in transit [24,28,51].
- Resistance Education: Crucially, the concordance rate was observed to decrease to 87% in pretreated patients [28,57]. Within our taxonomy, this delta is interpreted as “resistance education,” where prior systemic therapies have induced functional adaptations within the C-TAC population that are not yet reflected in the broader radiological picture [24,28,32,57]. This validates the use of C-TACs for real-time monitoring of therapeutic resistance [4,24,28].

### 7.2. Perioperative Stratification in Thoracic Oncology: The Sawabata Model

A cornerstone of this review is the evidence provided by Sawabata et al. (2024) regarding the predictive power of Circulating Tumor Emboli (CTE) in Non-Small Cell Lung Cancer (NSCLC) [19]. This research moves beyond prognosis alone toward a candidate framework for adjuvant therapy selection that will require prospective validation [19].

- The RFS Differential: In this study of 128 NSCLC patients, those harboring perioperative heterotypic clusters (strictly termed CTE) showed a substantial benefit from adjuvant chemotherapy, with a 71.8% 2-year Recurrence-Free Survival (RFS) [19].
- Identifying Futility: Conversely, patients who were CTE-positive but did not receive adjuvant treatment showed an RFS of only 36.3% [19]. Notably, patients without these clusters did not appear to derive a significant benefit from systemic therapy in this cohort. These findings are consistent with the hypothesis that CTE could help identify higher-risk patients who may be more likely to benefit from intensified perioperative intervention, a hypothesis that requires prospective confirmation [13,19].

### 7.3. Addressing Phenotypic Diversity: The Small-Cell Size Paradigm

The final pillar of our empirical validation concerns the necessity of size-independent detection, as highlighted by the work of Wen et al. (2025) in bladder cancer [59].

- The Small CTC Indicator: Traditional liquid biopsy platforms often focus on large, epithelial-positive cells [13,58]. However, Wen et al. identified that “small-size” CTCs—which are frequently missed by size-based enrichment—are critical predictors of recurrence in high-risk non-muscle invasive bladder cancer (NMIBC) [59].
- Validation of the Elemental Unit: Within our taxonomy, these small cells are categorized as part of the Elemental Unit (CTCs) [1,42]. Their clinical significance underscores the need for marker-independent and size-independent detection technologies, such as aneuploidy-based enrichment, to ensure that the most aggressive “seeds” of metastasis are not overlooked during clinical screening [13,25,42].

By synthesizing these disparate yet complementary lines of evidence, Table 2 demonstrates that the cTME/C-TAC hierarchy is not a theoretical abstraction but a clinically validated substrate for precision oncology [1,13,19,24,28]. The integration of these findings allows for a transition toward “Total cTME Profiling,” where genomic insights, functional dynamics, and physical stratification converge to guide the next generation of cancer care [1,4,12].

## 8. Technical Limitations and Platform Comparative Analysis: The Biophysical Divide

The clinical realization of the proposed cTME/C-TAC taxonomy is fundamentally contingent upon the technological substrate used for cellular isolation and characterization [1,13,24]. For over two decades, the primary hurdle in standardizing liquid biopsy results has been an inherent “analytical bias” introduced by traditional isolation methodologies [4,42], which often prioritize ease of use over biological inclusivity. To move toward a “Total cTME Profiling” approach, we must critically evaluate the biophysical and molecular limitations of current platforms [1,12,13], specifically addressing why marker-dependent systems have inadvertently created a diagnostic blind spot regarding the most aggressive executors of metastasis [4,13].

### 8.1. The EpCAM Paradox: Limitations of Marker-Dependent Capture

The CellSearch^®^ system, currently the only FDA-cleared platform for CTC enumeration, serves as the historical benchmark for the field [13,58]. However, its operational dependency on the expression of the Epithelial Cell Adhesion Molecule (EpCAM) creates a profound ontological oversight in the context of advanced malignancy [4,13,42].

- Epithelial–Mesenchymal Transition (EMT): It is now well-established that during the metastatic cascade, the most aggressive tumor cells often undergo EMT [34,43,44,45,60], a process characterized by the partial or complete loss of epithelial markers, including EpCAM and cytokeratins [4,34,44,45]. These cells instead acquire mesenchymal or cancer stem cell-like phenotypes that favor motility, immune evasion, and survival in the hostile circulatory environment [43,44,60]. Consequently, EpCAM-based systems systematically overlook the very subsets of the C-TAC population that are most likely to drive recurrence and therapeutic resistance [4,24,42,43].
- The “Invisible” Supporting Niche: The C-TAC framework relies on the detection of non-malignant supporting cells, such as Cancer-Associated Fibroblasts (CAFs) and Circulating Tumor-Derived Endothelial Cells (CTECs) [1,2,24]. By definition, these supporting cells lack epithelial markers; CAFs are of mesenchymal origin, and CTECs are endothelial derivatives [2,24,25,26,27]. Relying on marker-dependent capture renders these essential components of the “mobile niche” invisible, thereby preventing a comprehensive analysis of the circulating ecosystem [1,24,42].

### 8.2. SE-iFISH and the Shift to Aneuploidy-Based Enrichment

To resolve the limitations of protein-based capture, we advocate for a transition toward marker-independent technologies, exemplified by the SE-iFISH (Subtraction Enrichment and Immunofluorescence in situ Hybridization) platform [13,42]. This approach shifts the diagnostic focus from transient phenotypic markers to stable genomic abnormalities [25,42].

- Subtraction Enrichment (Negative Selection): Unlike positive selection, which risks missing cells with low marker expression, SE-iFISH utilizes a “negative list” approach [48]. By immunomagnetically depleting leukocytes (CD45+) and other common blood components, the system preserves the entire spectrum of rare cells within the CRC (Circulating Rare Cells) tier, regardless of their epithelial or mesenchymal status [24,42].
- Aneuploidy as a Stable Hallmark: Chromosomal instability, manifested as aneuploidy (specifically of chromosome 8), is a near-universal hallmark of malignant solid tumor cells [25]. By utilizing centromere probes for chromosome 8, SE-iFISH allows for the definitive identification of tumor cells and aneuploid CTECs [25]. This genomic stability ensures that the Elemental Unit (CTC) can be accurately tracked across the entire spectrum of therapeutic response and clonal evolution [13,25,42].

### 8.3. The Physics of “Small-Size” CTCs and Microfiltration Bias

The biophysical properties of C-TACs, particularly their size and deformability, present a significant challenge to traditional enrichment techniques [4,42]. Many marker-independent systems utilize size-based microfiltration, assuming that CTCs are significantly larger than the surrounding hematological cells [13,42].

- The Size Distribution Paradox: Recent clinical data has identified a critical population of “small-size” CTCs (≤5 μm) [59]. These cells, which are characterized by a high nuclear-to-cytoplasmic ratio and aggressive metastatic potential, are often missed by standard 8 μm microfilters [42,59]. In high-risk non-muscle invasive bladder cancer (NMIBC), these small cells have been identified as superior prognostic indicators compared to traditional large-cell enumeration.
- Taxonomic Necessity for Size-Invariance: Within the cTME framework, it is imperative that capture technologies remain size-invariant [1,48]. Platforms that rely on centrifugation or subtraction enrichment (like SE-iFISH) are better suited to capturing these small-cell variants, ensuring that the most aggressive “seeds” of metastasis are not inadvertently discarded during the filtration process [24,42].

### 8.4. Fluid Shear Stress (FSS) and Cluster Integrity Preservation

The physical survival of Circulating Tumor Emboli (CTE) within the systemic environment is governed by the laws of fluid mechanics [3,16,41]. The cTME is a hostile mechanical landscape where the FSS in peripheral arteries can reach 15–20 dyn/cm^2^ [3,41], a force sufficient to induce physical fragmentation or apoptosis in solitary cells [3,16,41].

- The “Physical Shield” of the Cluster: Multicellular aggregates (CTE) utilize their collective density to protect internal malignant cells from these mechanical forces [3,16,18,41]. This shielding is a core functional attribute of the “mobile niche” [1,18].
- Technological Disruption: Many microfluidic platforms utilize high-velocity flow and narrow channels to increase capture efficiency [13,42]. However, these high-energy environments can physically disrupt fragile heterotypic clusters, leading to the false reporting of single CTCs when aggressive aggregates were actually present [16,42].
- Optimizing for Structural Integrity: To accurately profile the C-TAC population, capture methodologies must prioritize the preservation of cluster integrity. Gentle sedimentation and low-pressure enrichment protocols are essential for maintaining the physical associations between malignant cells and their host-derived partners, such as the neutrophils that “escort” the tumor cells through the circulation [42,46,51].

### 8.5. Synthesizing the Divide: Toward Total cTME Profiling

The technical divide between marker-dependent and marker-independent platforms is ultimately a divide between a static prognostic snapshot and a dynamic functional readout [4,13,42]. To achieve “Total cTME Profiling,” the field must move beyond the “EpCAM trap” [4,13] and adopt platforms that can simultaneously isolate the entire C-TAC ecosystem—including small-size cells [59], EMT-positive cells [43,44,45,60], and heterotypic clusters [18,19]—while preserving their biological and physical state [1,24,42]. By integrating these biophysically robust capture methods with the proposed taxonomic hierarchy [1], we provide clinicians with a clearer, more accurate roadmap for real-time personalized cancer management [4,13,24,42].

## 9. Discussion: Navigating Economic, Regulatory, and Clinical Barriers

The architectural implementation of the cTME/C-TAC taxonomy is not merely a theoretical exercise in biological classification [1]; it represents a fundamental paradigm shift intended to guide real-time therapeutic decision-making in the clinic [1,4]. However, the transition from a research-based, single-analyte liquid biopsy to a comprehensive “Total cTME Profiling” approach requires navigating a complex landscape of economic, regulatory, and clinical challenges [4,13]. This section addresses the structural barriers that must be overcome to realize the full potential of this unified roadmap for precision oncology [4,13,24].

### 9.1. The Economic Paradox: High-Resolution Diagnostics vs. Healthcare Sustainability

One of the primary hurdles in the clinical adoption of multi-dimensional profiling is the perceived increase in diagnostic costs [4,13,42]. Transitioning from traditional, single-cell enumeration to a multi-modal analysis—incorporating ctDNA, C-TAC-based CRP, and EV-mediated niche signatures—involves significant upfront investments in sophisticated bioinformatics and diverse laboratory processing streams [1,9,24,55,56].

- Cost-Effectiveness through Precision: Despite the higher analytical costs, this approach must be viewed through the lens of long-term healthcare sustainability [4,13]. If validated, the ability to avoid futile, high-cost systemic therapies through Chemo-Response Profiling (CRP)—for which a 97% concordance in therapy-naïve patients has been reported in a preliminary study—could represent a meaningful potential saving for healthcare systems [28,32,34]. By identifying patients who will not benefit from a specific regimen before treatment begins, we can mitigate both the financial burden and the physical toxicity of ineffective care [4,9,28,57].
- Resource Allocation: Establishing a reimbursement framework that prioritizes “functional” over “static” data is essential [4,13]. Regulatory bodies must recognize that the integrated cellular and genomic readout provided by the cTME hierarchy offers a level of predictive certainty that justifies the initial diagnostic expenditure [1,13,22].

### 9.2. Regulatory Standardization and the Global Liquid Biopsy Roadmap

A critical structural challenge identified throughout this review is the need for a standardized classification framework to support reproducible clinical reporting across institutions [4,13]. Standardizing this unified taxonomy is a clinical imperative that requires global cooperation between research institutions and regulatory agencies [13].

- Decoupling Environment from Aggregate: As we move toward the era of precision oncology, it is essential that clinical trial protocols strictly decouple the systemic ecosystem (cTME) from the aggressive physical clusters (CTE/CTM) [1,18,19]. This terminological clarity will allow for the cross-platform comparability of results, ensuring that a “high-risk” designation in one center corresponds to the same biological reality in another [4,13].
- The Need for Marker-Independent Capture: From a regulatory standpoint, standardizing capture protocols is essential for multi-center trials [13,42]. The current reliance on epithelial markers has limited the analytical scope of liquid biopsy [4,13,42]. Moving forward, regulatory approval should prioritize technologies—such as aneuploidy-based enrichment—that capture the entire C-TAC population, including small-size CTCs and EMTs, ensuring that the most aggressive executors of metastasis are consistently reported [24,42,59].

### 9.3. Clinical Integration: Transforming the Surgical Paradigm in Thoracic Oncology

For the oncologist, the integration of cTME data into the perioperative window represents the ultimate clinical application of this taxonomy [1,4,13]. The ability to track the real-time evolution of a patient’s “flowing microenvironment” allows for a level of strategic planning previously unavailable [4,9,13].

- Refining Adjuvant Strategy: The research in Non-Small Cell Lung Cancer (NSCLC) provides an illustrative, though still preliminary, model for this integration [19]. By identifying perioperative CTE, surgeons can move beyond traditional pathological staging to include functional risk markers [4,19]. The dramatic survival benefit observed in CTE-positive patients receiving adjuvant chemotherapy—71.8% RFS versus 36.3% in untreated cases—provides strong preliminary evidence supporting the future exploration of liquid biopsy results for formal surgical consensus guidelines, contingent upon validation in larger, prospective multi-center cohorts [13,19].
- Predicting the “Soil” and the “Seed”: A “Total cTME Profile” allows the clinician to monitor both the preparatory signals and the functional executors [1]. Utilizing EVs to monitor the establishment of the Pre-Metastatic Niche (PMN) offers a window into the future site of recurrence, while C-TAC-based CRP offers a real-time roadmap for therapeutic selection [1,11,24,28,35,36,39,55,56]. This dual-monitoring strategy enables a truly proactive approach to managing minimal residual disease (MRD). Looking forward, AI-assisted integration of these complex multimodal datasets will be essential to synthesize multi-omic cTME profiles into actionable clinical blueprints. Such computational models hold immense potential for expanding the taxonomy’s utility into advanced downstream applications, particularly for real-time immunotherapy tracking and longitudinal response evaluation [33,61].

In conclusion, navigating the barriers to adoption requires a multifaceted effort to align economic reality with biological insight. By adopting this unified taxonomy, the oncology community can advance toward a new era of real-time, personalized cancer management [1,4,13,24].

## 10. Conclusions: Toward “Total cTME Profiling”

The transition of liquid biopsy from a rudimentary single-analyte counting tool to a sophisticated, multi-dimensional biological roadmap represents one of the most significant clinical imperatives in modern oncology [4,13]. Throughout this comprehensive review, we have established that the true transformative potential of liquid biopsy requires a structured, multi-tiered classification framework that can accommodate its expanding biological and technological scope [1,13]. Without such a framework, the integration of diverse data streams—from the broad systemic ecosystem to localized physical cellular clusters—into actionable clinical platforms remains fragmented [4,13]. By proposing a rigorous and unified taxonomy that strictly decouples the superordinate Circulating Tumor Microenvironment (cTME) from the aggressive physical aggregates strictly classified as Circulating Tumor Emboli (CTE/CTM), we provide the oncology community with the intellectual and biological clarity required for the next generation of precision medicine [1,18,19].

This framework is not merely a semantic refinement but the essential foundation for a new diagnostic paradigm we define as “Total cTME Profiling” [1]. In this advanced model, the liquid biopsy ceases to be a static snapshot of disease and becomes a dynamic, real-time synthesis of disparate but complementary biological datasets [4,13,42]. The genomic and molecular cues provided by ctDNA and EVs—the latter enriched via innovative pH-sensitive technologies such as the D-S PHLIP system—offer critical insights into the clonal evolution of the primary tumor and the preparatory signals governing the formation of pre-metastatic niches [35,36,55,56]. Simultaneously, the functional readout of viable C-TACs, as suggested by the 97% concordance rate of Chemo-Response Profiling (CRP) reported in therapy-naïve patients, may offer a functional evaluation of therapeutic efficacy that complements genomic mapping, pending prospective validation [28,32,34].

Furthermore, the integration of physical stratification through the detection of high-risk CTE and the elusive small-size CTCs (≤5 μm) represents a critical advancement in patient risk assessment [45,48]. Particularly in the perioperative setting of malignancies such as Non-Small Cell Lung Cancer (NSCLC), identifying these aggressive executors of metastasis allows clinicians to move beyond conventional pathological staging and implement adjuvant strategies tailored to the unique biological risk profile of the individual patient [4,19]. The clinical imperative for this taxonomy is underscored by its ability to distinguish between patients who will derive a massive survival benefit from systemic intervention and those for whom such treatment would be futile [13,19,28].

In conclusion, adopting this unified taxonomy is more than a semantic exercise; we propose it as a conceptual roadmap that may help guide the future development of personalized cancer management, while recognizing that its clinical value remains to be established through prospective study [1,4,13]. By recognizing the cTME as the superordinate systemic ecosystem and C-TACs as its functional executors, we move toward a future where every therapeutic decision is guided by the multidimensional reality of the tumor’s “flowing” microenvironment [1,24]. This shift to “Total cTME Profiling” ensures that we no longer treat cancer as a static entity, but as a dynamic biological process that can be monitored, predicted, and ultimately controlled [4,13,42]. As we advance, this framework will empower clinicians to deliver the right treatment to the right patient at precisely the right time, transforming the liquid biopsy into the cornerstone of 21st-century precision oncology [1,4,13,24].

## Figures and Tables

**Table 1:** Systematic Comparison and Hierarchical Relationships of Key Liquid Biopsy Components.

Concept	Scope of Components	Core Functional Role	Clinical Application Example	Conceptual Hierarchy
cTME	Systemic ecosystem (Cellular, non-cellular, biophysical) [1,13]	Systemic regulation of metastatic cascade [1,36,55,56]	Multi-modal prognostic profiling * [1,12,13]	Superordinate tier (highest inclusive concept) [1]
CRC	Broad non-hematological rare cell spectrum [2,25,49]	Pan-cellular diagnostic screening [13,49]	Early detection research * [4,13]	Highest cellular category [13,24]
C-TACs (Circulating Tumor-Associated Cells)	Heterogeneous collectives (malignant + host cells) [24,26,27,46]	Functional execution of metastasis [24,28,32,57]	Chemo-response profiling (CRP) * [28]	Functional subset of cTME [1,24]
CTE/CTM (Circulating Tumor Emboli)	Multicellular aggregates (homotypic/heterotypic) [16,17,19]	Aggressive seeding via a “mobile niche” [3,18,41]	Adjuvant efficacy predictor * [19]	Aggressive subset of C-TACs [17,18,19]
CTCs (Circulating Tumor Cells)	Malignant cells only (solitary/small clusters) [43,49]	Metastatic “seed”; basic prognostic indicator [43,58]	Traditional cell enumeration (e.g., CellSearch^®^) [13,58]	Elemental unit of C-TACs [1,43]

Abbreviations: cTME, Circulating Tumor Microenvironment; CRC, Circulating Rare Cells; C-TACs, Circulating Tumor-Associated Cells; CTE/CTM, Circulating Tumor Emboli/Microemboli; CTCs, Circulating Tumor Cells. Specific components of the cTME include molecular messengers (ctDNA, EVs) and biophysical factors (fluid shear stress, pH gradients). C-TACs encompass CTCs and non-malignant partners such as CAFs and CTECs. Rows are ordered hierarchically from the most inclusive system to the elemental unit. * Cautionary Note on Clinical Applications: The clinical applications listed—such as the CRP concordance and predictive utility for adjuvant therapy—are derived from preliminary, proof-of-concept studies. These promising frameworks are hypothesis-generating and require independent prospective multi-center validation before adoption as routine clinical standards.

**Table 2:** Summary of Major Clinical Evidence Supporting the cTME/C-TAC Taxonomy.

Study	Cohort	Key Finding	Clinical Significance
Crook et al. [28]	n = 5090	97% CRP concordance in therapy-naïve patients [28].	[Proof-of-Concept] Potential functional cellular unit for real-time therapy prediction [24,28]. Derived from retrospective data; requires independent prospective validation.
Sawabata et al. [19]	n = 128 (NSCLC)	CTE-positive status associated with adjuvant chemotherapy benefit [19].	[Emerging Clinical Utility] Candidate predictive biomarker for perioperative stratification [19]. Derived from single-center retrospective data; awaits multi-center prospective validation.
Wen et al. [59]	N/A (Bladder Cancer)	Small-size CTCs (≤5 μm) significantly predict recurrence [59].	[Preliminary/Indirect Support] Highlights the clinical necessity for size-independent detection within the CTC elemental unit [42,59]. Observational data from a distinct tumor type (NMIBC).

This table highlights empirical clinical validation for transitioning from simple enumeration to functional profiling. Crucial Limitation Note: All listed associations currently derive from a limited number of single-center, retrospective, or observational studies. These findings must be regarded as preliminary and hypothesis-generating. None has yet been established in independent, multi-center, prospectively designed clinical validation cohorts, and this table should be interpreted accordingly.

## Data Availability

No new data were created or analyzed in this study.
